# ^36^Cl/Cl Accelerator-Mass-Spectrometry Standards: Verification of Their Serial-Dilution-Solution Preparations by Radioactivity Measurements

**DOI:** 10.6028/jres.098.043

**Published:** 1993

**Authors:** R. Collé, Joylene W. L. Thomas

**Affiliations:** National Institute of Standards and Technology, Gaithersburg, MD 20899-0001

**Keywords:** accelerator mass spectrometry (AMS), beta counting, chlorine-36, liquid scintillation (LS), measurements, proportional counting, radioactivity, standards

## Abstract

A consortium of accclerator-mass-spectrometry (AMS) laboratories recently prepared a series of ^36^Cl/Cl isotopic ratio AMS standards by an eight-step serial gravimetric dilution scheme. Of the resulting nine solutions, only the latter six could be assayed by AMS to confirm the gravimetric dilution factors. This paper provides the results of relative radioactivity measurements on the first four solutions to verify the first three dilution factors. The fourth solution was the only dilution capable of being directly measured by both AMS and radionuclidic metrology of ^36^Cl, and therefore its assay by radioactivity counting was deemed of considerable importance. Assays were performed by 4π*β*^−^ liquid scintillation (LS) counting of gravimetric aliquots of the solutions, with confirmatory measurements by 2π*β*^−^ gas-flow proportional counting of gravimetrically-prepared solid sources. The radioactivity measurements on the fourth solution were complex and difficult because of the conflicting combination of a low activity concentration (0.036 Bq · g^−1^) and high salt content (146 mg NaCl per g of solution). These conditions necessitated independent studies of the ^36^Cl LS efficiency as a function of NaCl loading in the LS cocktails and of the feasibility of LS counting of precipitated samples, both of which are also reported here. The results of the radioactivity measurements confirmed the dilution factors for the first three solutions to absolute differences of about 1%, and that for the fourth solution to about 1% to 2%. The overall uncertainties for these verification measurements, at a relative three standard deviation uncertainty interval, were of comparable magnitude, i.e., in the range of ±1% to ±2% for the first three solutions and roughly ±3% for the fourth solution.

## 1. Introduction and Overview

Measurements of ^36^Cl by accelerator mass spectrometry (AMS) significantly contribute to research in a variety of disciplines within the geologic, hydrospheric, atmospheric and cosmic sciences [1], and references therein, as well as in other applied areas. For example, such AMS measurements recently were used to evaluate the neutron exposure dosimetry models for the Hiroshima and Nagasaki atomic bombings [2].

The importance of these measurements demands the availability of reliable and accurate ^36^Cl/C1 isotopic standards both for calibrations between laboratories and for internal controls within a laboratory.

A series of ^36^Cl/Cl AMS standards were recently developed and prepared by a consortium of AMS laboratories [1]. The six AMS standards consist of solutions containing 145.6 mg NaCl per g of solution and have atom ratios of 3.254 × 10^−10^, 5.003 × 10^−11^, 1.000 × 10^−11^, 5.000 × 10^−12^, 1.600 × 10^−12^, and 5.000 × 10^−13^. The solutions were prepared by serial gravimetric dilutions of a ^36^C1 solution standard, viz., National Institute of Standards and Technology (NIST) Standard Reference Material SRM 4943. The gravimetric dilution factors were estimated to have uncertainties of less than ± 1% in terms of a relative standard deviation. The dilutions were performed at the University of California at San Diego (UCSD), and were subsequently checked in three separate experimental trial runs using the University of Rochester AMS facility. In each case, the absolute percent difference in the measured ^36^C1/C1 atom ratio compared to the expected atom ratio, based on gravimetric dilution factors, was better than 2%, The measured atom ratios for the new AMS standards were also in excellent agreement with older UCSD ^36^C1/C1 standards [1].

In preparing the six new AMS standards, the original stock solution (NIST SRM 4943) underwent an eight-step serial dilution. For additional details, refer to Fig. 1 in [1]. The results of the first three dilutions are summarized in Table 1 where solution A is the SRM stock solution, solution B is a dilution of A, solution C is a dilution of B, and solution D is a dilution of C. Solution D is the first aforementioned AMS standard, and was further used in five dilutions to prepare the remaining five AMS standards. It is the AMS standard of largest ^36^C1/C1 atom ratio and the least dilution capable of being directly measured by AMS.

The experimental design and plan for the preparation of the AMS standards originally envisaged having NIST perform relative radioactivity measurements on the four solutions of Table 1 to verify the first three gravimetric dilution factors [1]. This paper provides the results of these verification measurements.

Measurements of solutions A, B, and C by 4π*β*^−^ liquid scintillation (LS) counting techniques to within uncertainties of ±1% to ±2% at a relative three standard deviation uncertainty interval appeared to be straightforward. The measurement of solution D, however, required more laborious and heroic efforts. Inasmuch as solution D was the only dilution of the series capable of being directly measured by both AMS and radioactivity counting, the expense of considerable effort in trying to assay it with comparable accuracy, if possible, was deemed to be a worthwhile enterprise.

The measurement of solution D by LS counting was problematic because of the combination of a relatively low activity concentration (approximately 0.036 Bq · g^−1^) in conjunction with a relatively high NaCl concentration (146 mg NaCl per g of solution). The low activity concentration required loading LS cocktails with sample sizes as large as possible to enhance the net counting rates above background. Even with a high ^36^C1 LS detection efficiency of nearly 99%, the net LS count rate per g of solution D was expected to be only about 3% of background. Hence, even a 5 g LS counting sample would result in a net rate of around 15% of background. In addition, it was subsequently discovered that the dissolved NaCl in these salt solutions (at 146 mg NaCl g^−1^) began to precipitate out of cocktails prepared with even less than 0.4 g of sample and 15 g of conventional, commercially-available scintillation fluids known to have high salt loading capacities. Even increasing the aqueous phase content in the LS cocktails (with blank water) to 10% only allowed sample aliquots of about 0.75 g (i.e., 110 mg NaCl) before salt precipitates began to form. With any further increases in aqueous phase content, in which the cocktails went from clear solutions to translucent and opaque counting gels, the NaCl solubility in the cocktails actually decreased with increasing water content. At the same time, the effects of such large NaCl loadings on the LS efficiency for ^36^C1 were largely unknown. Therefore, prior to initiating the intended verification measurements of the AMS standard serial dilutions, it was necessary to conduct several preliminary investigations of the ^36^Cl LS efficiency as a function of NaCl cocktail loading and of the feasibility of LS counting of precipitated samples. This paper then also provides the results of those studies.

Lastly, this paper describes the attempts that were made to perform confirmatory measurements by low-background, 2π*β*^−^ flow-proportional counting of evaporated solid sources prepared from the AMS standard solutions. From the onset, it was considered that these solid source measurements would at best only serve as less accurate, but independent confirmations of the LS results. As for the LS measurements, the *β*^−^ proportional counting was similarly problematic for solution D because of the rather massive NaCl concentration. The source preparation requirements and attendant restrictions for the two cases were, in fact, comparative. The evaporated counting sources had to be optimized between using large aliquots to increase the net *β*^−^ count rates and small aliquots to decrease self-absorption and scattering *β*^−^ count rate losses.

It should be noted, for completeness, that other ^36^C1 radioactivity assay techniques were also considered. No others within the capability of the NIST Radioactivity Group, however, appeared feasible. The most viable alternative candidate was by direct Cerenkov counting of the solutions, but it too appeared to be impracticable. The ^36^C1 *β*^−^ spectrum is insufficiently energetic (maximum *E_β_−*=709.3 keV; average *E_β_−* = 251.2 keV) to produce a large Cerenkov radiation yield. It was estimated that the ^36^C1 detection efficiency by Cerenkov counting was likely to be less than 3% to 5% under even the most optimum sample conditions [3]. All photon spectrometry techniques also had to be excluded since ^36^C1 primarily decays directly to the ground state of ^36^Ar (98.1% *β^−^*) with weak electron-capture (1.9% EC) and *β^+^*-decay (0.0017%) branches to the ground state of ^36^S without the emission of any gamma rays [4]. The x rays accompanying the EC decay and the 511 keV annihilation radiation from *β^+^* decay interactions are insubstantial for assaying purposes at the desired ±1% to ±2% relative three standard deviation uncertainty interval.

## 2. Measurement Methods, Results, and Discussion

### 2.1 Liquid Scintillation Measurements

In assaying the four solutions under test (Table 1), the LS counting sources, in all cases, consisted of an appropriate aliquot of one of the solutions and approximately 15.1 g of Beckman^1^ “Ready Safe,” a polyarylalkane (i.e., an alkylated biphenyl)-surfactant-based scintillation cocktail, contained within nominal 22 mL, glass LS vials. The cocktail was selected in part because of its reported high holding capacity for aqueous salt solutions [5], as well as being environmentally safe (i.e., non-toxic, non-flammable, and bio-degradable). Some of the sources also contained respective proportions of a blank doubly-distilled water solution and a carefully prepared blank NaCl solution having a concentration of 145 mg NaCl per g of solution. The blank water and NaCl solutions were used to vary the NaCl content in some of the counting sources, to match sample compositions (and hence sample quenching) for the different sources prepared from the four solutions, and to prepare matched blanks of nearly identical composition for background subtractions.

All of the sample aliquots used to prepare the counting sources were determined gravimetrically with an estimated uncertainty, corresponding to an assumed standard deviation, of ± 10 μg to ± 30 μg. For sample aliquots ranging from about 30 mg to 60 mg for solution A to greater than 5 g for solution D, the relative standard uncertainties in the sample mass measurements thus are about 0.05% to less than 0.001% in general, and about 0.1% in worst cases, after appropriate gravimetric measurement air-buoyancy corrections, and considering the internal balance-weight uncertainties, and typical mass measurement precision.

The sources were measured in two LS counting systems: (1) a Beckman LS7800 model LS counter equipped with two Hamamatsu R331-05 photomultiplier tubes operating in a coincidence mode, a logarithmic pulse amplifier coupled to an analog-to-digital converter (ADC) for spectral pulse-height analysis, and an external 137Cs source for Compton-edge (Horrocks number) quench monitoring [6]; and (2) a Packard Tri-Carb 2500TR LS analyzer employing two matched high performance photomultiplier tubes also operating in coincidence, but with linear amplification for ADC pulse-height spectral analysis, and with an external ^133^Ba source that provided an instrument-provided, “transformed Spectral Index of the External Standard (*tSIE*),” quench indicating parameter [7].

Based on theoretical modelling predictions using the CIEMAT/NIST ^3^H efficiency tracing method calculations [8–10], the LS counting efficiency for ^36^C1 in unquenched samples was expected to be about 98% to 99%. This overall efficiency results from a nearly 100% efficiency for the 0.981 *β*^−^-decay branch and with a reasonably high (perhaps 25% to 75%) efficiency for the 0.019 electron capture (EC) branch. Refer to Table 2.

The LS efficiency, as well as cocktail stability, for samples quenched with large quantities of NaCl was however unknown, and required a preliminary study.

#### 2.1.1 LS Efficiency as a Function of NaCl Loading

To investigate the effect of NaCl loading on the ^36^C1 LS counting efficiency, a series of counting sources with varying NaCl content were prepared with known amounts of ^36^C1 activity from NIST SRM 4943 (solution A) and with the blank water and NaCl solutions (see Table 3). Each of eleven sources contained from about 20 mg to 65 mg of solution A (about 220 Bq to 720 Bq). The first two sources (SA1 and SA2 in Table 3) contained no additional blank solutions, and hence had minimal NaCl (≤ 0.01 mg) and very low total aqueous content (<0.3% water by mass). The next two (SA3 and SA4) were prepared with about 1.2 g and 1.5 g of blank water to a nominal 9% to 10% aqueous content. These two also had minimal NaCl. The next seven (SA5 through SAH) were prepared with varying combinations of blank water and NaCl solutions to give increasing NaCl contents (from about 45 mg to 230 mg NaCl) and with a nearly constant 9% to 10% total aqueous content. The samples with NaCl content up to about 110 mg were clear. The next two (SA8 and SA9) appeared clear at first, but upon settling for a few days began to exhibit a trace of salt precipitates. The last two samples (SA10 and SAH) had large salt precipitates.

Each of the samples along with matched blanks for each sample composition were measured four times over two days with the Beckman LS counter. The counting results are summarized in Table 3 in terms of the calculated ^36^C1 LS efficiency (mean of the four measurements), the relative standard deviation of the mean *s*_m_, and the average Horrocks number for each sample. The Horrocks number (H#) is a quench indicating parameter that is based on the downward spectrum shift of the Compton edge of an external ^137^Cs standard with increasing sample quenching. The parameter corresponds to the spectral channel number shift between the sample and an unquenched blank sample.

As indicated in Table 3, for the first two samples (SA1 and SA2) containing minimal NaCl and less than 0.3% water, the quench parameter was about *H#* = 65 and the efficiency was approximately 0.986 for the mean of the two. With increasing water content to about 9% to 10% (samples SA3 and SA4), *H#* increased to approximately 95 with a small or almost negligible change in efficiency (mean = 0.982). With increasing NaCl content from the minimal quantities up to about 110 mg (samples SA3 through SA7), the values of *H#* remained at about 95 and the efficiencies were virtually identical. With further increases in NaCl content in which precipitates formed in the counting sources (samples SA8 through SAH), the efficiencies began to slightly decrease. At the same time, the measurement precision on these precipitated samples considerably worsened as evidenced by the large differences in efficiency for samples of very similar composition and by the much larger values of *s*_m_ for measurements on even the same samples, The apparent irregularities in the efficiencies as a function of this NaCl loading in precipitated samples will be discussed at length in Sec. 2.1.3. Interestingly, the quench parameters for these samples actually decreased (from *H#* = 95 to *H#* = 72) with increasing NaCl and increasing precipitation in the samples. It is conjectured that this effect arose because with the increasing precipitation of the salt out of the cocktail mix, the scintillator phase itself became more transparent and contained less of the NaCl to quench the samples. The most surprising overall result however was the relatively high efficiency values (> 95%) that were obtained with these large NaCl loadings and moreover that they were obtained even in samples with substantial precipitate formations.

Spectra for these various samples as a function of NaCl loading are discussed in Sec. 2.1.3.

The results of this investigation (Table 3) clearly established the bounds for the composition of LS counting sources that would not result in the formation of precipitates and that would provide reasonably low quenching and high detection efficiency with good measurement precision. The experimental design for the assays of solutions A, B, and C were based on these results. The low activity concentration for solution D, as described in the Introduction (Sec. 1), precluded a similarly based assay procedure.

#### 2.1.2 LS Counting of Solutions A, B, and C

Three LS counting sources were prepared for each of the solutions A, B, and C such that each source contained approximately 110 mg NaCl and 9% water in 15 g of scintillator (see Table 4). The sources were prepared with varying aliquots of the solutions (ranging from 32 mg to 43 mg for A; 260 mg to 720 mg for B; and about 750 mg for C) and with appropriate quantities of the blank water and blank NaCl solutions. These nine sources (along with three others prepared earlier; namely SA1, SA2, and SA7) and matched counting blanks of nearly identical sample composition were measured five times on both the Beckman and the Packard LS counting systems.

The counting results are tabulated in Tables 4 and 5, respectively. The tables provide the mean counting rates per unit mass of solution for each source, computed statistics for the measurement precision, sample quenching parameters, derived or assumed LS counting efficiencies, and the resultant activity concentrations.

The Horrocks number *H#* was described previously. The Packard system’s quench indicating parameter *tSIE* is based on a mathematical analysis of the Compton spectrum and consists of a decreasing relative quenching scale in which unquenched samples correspond to *tSIE* = 1000.

The quench parameters *H#* (for the Beckman LS system) and *tSIE* (for the Packard) scale proportionally and are closely matched in all cases, excepting those for samples SA1 and SA2 which contained less water and which were recounted only for comparative purposes. The measurement precision both in terms of the relative standard deviation of the mean for the five measurements (*s*_m_) and in terms of the relative standard deviation for the assumed Poisson-distributed total statistical “counting error” (*s_p_*)^2^ varied appropriately with the respective activity concentrations in the three solutions. The activity concentrations in units of Bq · g^−1^ for solutions B and C were derived from the mean LS counting efficiencies for each counting system which were in turn obtained from the mean count rate concentrations for similarly matched samples and the known activity concentration of solution A (see Tables 4 and 5). Alternatively, as provided in Table 6, ratios of the mean counting rate concentrations for the various solution pairs A/B, A/C, and B/C can be directly compared to the reported gravimetric dilution factors.

The measurement uncertainties for these determinations are summarized in Table 7. The uncertainty analysis procedures used here follow the normal conventions of the NIST Radioactivity Group which for the most part are compatible with those adopted by the principal international metrology standardization bodies [11]. All individual uncertainty components are expressed in terms of estimated (experimental) standard deviations (or standard deviations of the mean where appropriate) or quantities assumed to correspond to standard deviations, irrespective of the method used to evaluate their magnitude. A combined or propagated uncertainty is expressed as an estimated standard deviation which is equal to the positive square root of the total variance obtained by summing all variance and covariance components, however evaluated, using the law of propagation of uncertainty for the specific mathematical function given by the model of the measurement procedure. By convention in this laboratory, the combined uncertainty is expanded by a factor of 3 to obtain an “overall” or expanded uncertainty which is assumed to provide an uncertainty interval having a high level of confidence of roughly 95% to 99%.

The results of these LS determinations for solutions A, B, and C were also subjected to extensive statistical tests on subsets of the data across a variety of variables: sample preparation order, sample composition, sample masses, sample replicates for a given solution, the timing and sequence of the LS measurements, and the LS measurement system used. These included χ^2^-tests of the homogeneity in subsets of the observed sample variances (across the variables), F-tests of the homogeneity in the various subset sample means, t-tests of differences between the various means, and tests of possible correlations and biases using analysis of variance (ANOVA) techniques with sequential two-way classifications of the variables. None of the tests indicated any statistically significant differences in any of the tested subset sample means or sample variances. The agreement in the results between the two LS counting systems as provided in Tables 4, 5, and 6 are representative.

The summarized results of these tables clearly verify and confirm the activity concentrations for solutions B and C, relative to solution A, to absolute differences of better than 1%; and they comparably verify and confirm the dilution factors for the A/B, A/C, and B/C solution pairs. The overall LS measurement uncertainties, at a three standard deviation uncertainty interval, as given in Table 7, are also of a comparable ±1% magnitude and are less than ± 2% in worst cases.

#### 2.1.3 LS Efficiency of Precipitated Samples

To investigate the feasibility of assaying solution D with precipitated LS samples, a series of precipitated-sample LS counting sources with increasing NaCl content were prepared as summarized in Table 8. The samples were prepared with about 15.1 g of scintillator and with known gravimetrically-determined aliquots of solution A (about 0.08 g to 0.1 g) and with varying blank water and blank NaCl solutions to vary the total NaCl loading. A similar set of blanks with closely matched sample compositions were also prepared for use in making background subtractions. All of the samples had a visible precipitate. The samples ranged from approximately 110 mg NaCl in 5.2% water with a very small salt precipitate that barely covered the bottom of the LS vial to 800 mg NaCl in 27% water with a massive salt precipitate that filled nearly one third of the LS vial’s volume. These loadings would correspond to solution D aliquots ranging from 0.75 g to 5.5 g. Recalling the discussion in the Introduction (Sec. 1), it was desirable to try to maximize the sample size to maximize the net LS count rates above background.

**Fig. 1:**
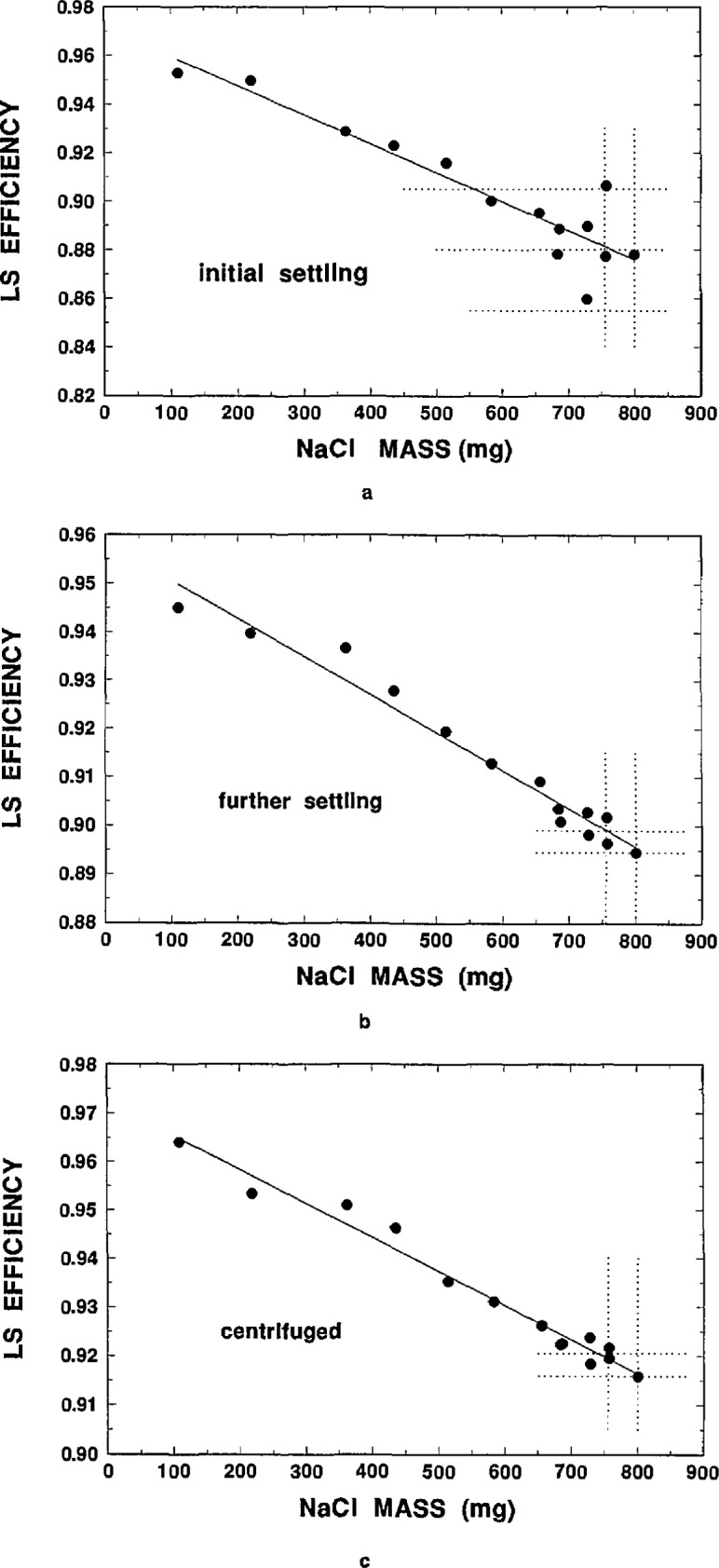
^36^Cl LS efficiency as a function of total NaCl loading in preeipitated-sample cocktails for three settling conditions: (a) “initial” after 2 d to 4 d; (b) “further” settling after 14 d to 16 d; and (c) for “centrifuged” samples.

Table 8 also contains LS counting results on these samples for two experimental trials. Both trials were based on five measurements of each sample over about 2 d with the Beckman LS system. These initial results are tabulated in terms of the mean ^36^C1 efficiency for the five measurements and the average *H#* quench parameter for each sample. The general trends indicated increasing sample quenching and decreasing efficiency with increasing NaCl content. The measurement precision however within the five measurements for a given sample was somewhat greater than typical LS measurement precision. It was also apparent that the efficiency as a function of total NaCl loading was not very systematic or regular (e.g., not exhibiting a monotonical functionality between the efficiency and NaCl content) particularly for samples greater than 650 mg NaCl. Subsequent measurements also revealed that the efficiency was a slowly varying function of the settling time. The efficiency, ϵ, as a function of NaCl mass, *m*, for three settling conditions are shown in Fig. 1: (a) are the data of Table 8 following about 2 d to 4 d of settling; (b) contains the results obtained after about 14 d to 16 d of settling; and (c) are the results for samples after undergoing centrifuging. The latter measurements were made since it seemed intuitive that centrifuged samples might approximate a steady-state settling condition. This intuition was proved to be mistaken.

All of the efficiency values of Fig. 1 are based on five replicate measurements with the Beckman system. As mentioned, the internal measurement precision within five measurements for a given sample was poor, with values of *s*_m_ ranging from typical LS measurement precision to over 1% in some cases. This can be compared (Table 3, for example) to previously observed values of *s_m_* for unprecipitated LS samples, in which *s*_m_ is typically better than 0.05% for a similar number of measurements. The irregularity in the efficiency as a function of NaCl mass is also readily observed in Fig. 1. The lines in each of the graphs of Fig. 1 correspond to χ^2^-minimized linear regressions fitted to the data, and even the slopes de/dm for the three settling conditions are substantially different. The fits are not intended to imply a theoretical significance, but were only made for comparison purposes. The three fitted slopes in Fig. 1 are d*ϵ/*d*m* = −0.119 g^−1^, −0.079 g^−1^, and −0.070 g^−1^, respectively, and have efficiency intercepts (extrapolated to zero NaCl mass) of *ϵ*_0_=0.972, 0.958, and 0.972. It must be emphasized however that all of these efficiency measurements were very temporal and dependent on the settling time for the samples.

**Fig. 2:**
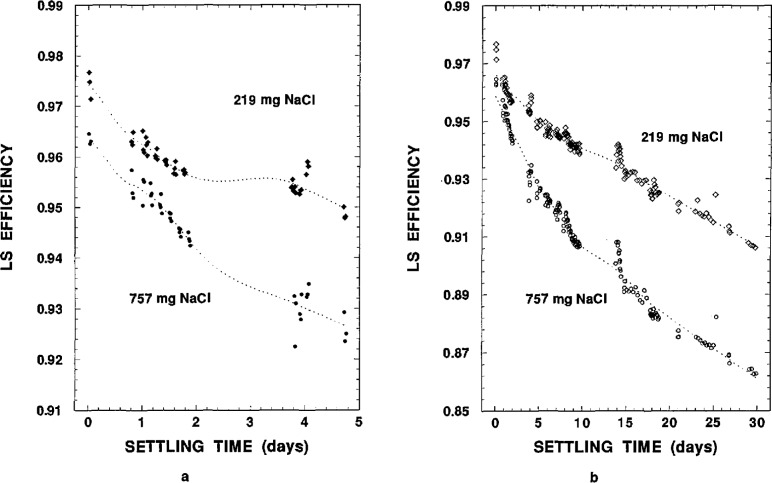
^36^C1 LS efficiency as a function of settling time for two precipitated-sample cocktails containing 219 mg and 757 mg NaCl. Measurements data for settling times (a) up to 5 d, and (b) up to 30 d. The dotted curves have no significance and are only meant to guide the eye.

Extensive subsequent measurements on these precipitated samples with settling times extending to over 102 d revealed additional unusual and inexplicable behavior. Figure 2 shows the efficiency *ϵ* as a function of settling time *t* for two sources containing 219 mg and 757 mg NaCl, displayed over two time scales: (a) from 0 d to 5 d and (b) from 0 d to 30 d. The observed wide variations in the efficiency for near replicate measurements with only small changes in settling time will be addressed below.

**Fig. 3:**
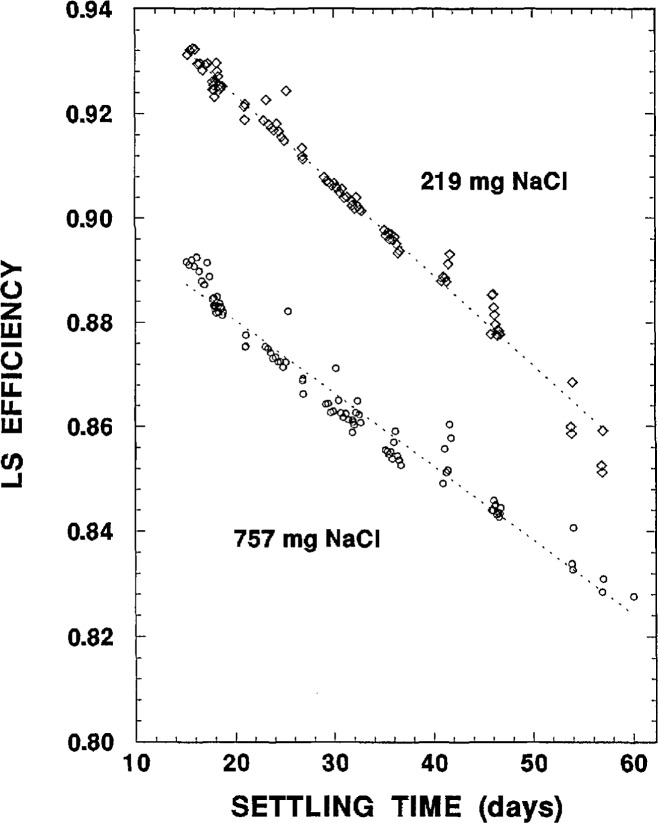
^36^C1 LS efficiency data for the two precipitated samples for settling times between 15 d and 60 d. The dotted lines are χ^2^-minimized linear regressions.

The more massive 757 mg NaCl source had an initially lower efficiency, as might be expected. For both sources, one can observe an abrupt decrease in efficiency of about 1% in 1 d (i.e., d*ϵ/*d*t* = 0.01 d^−1^). This rate of change gradually decreased over the next several days until the two slopes for the efficiency became somewhat constant (over the interval from about 5 d to 30 d) at about d*ϵ/*d*t*= −0.0017 d^−1^ for the 219 mg source and d*ϵ/*d*t* = −0.0026 d^−1^ for the 757 mg source. The less massive source not only had a smaller slope, but its transitory inflection point from its initial d*ϵ/*d*t* = 0.01 d^−1^ slope occurred earlier. These effects may be explainable in terms of the more massive source having a larger precipitate that settled more rapidly. The apparent nearly linear decreases in efficiency became even more constant after about 15 d of settling, although it now appeared that the more massive 757 mg source had a d*ϵ*/d*t* slope less than that for the 219 mg source. The efficiency data for settling times in the interval from 15 to 60 d are shown in Fig. 3, and demonstrate the near linearity in this region. Linear regressions (shown as dotted lines in Fig. 3) provided fitted slopes of d*ϵ/*d*t* = −0.00173 d^−1^ and −0.00141 d^−1^.

One may note that the efficiencies obtained with the centrifuged samples [Table 8 and Fig. 1(c)] corresponded to efficiencies that were also obtained after about 6 d or 7 d of settling.

Up to this time, *t*⪝60 d, except for the reversal in the relative magnitudes of the slopes for the two sources, the efficiency settling data seemed reasonably consistent and intuitively explainable.

**Fig. 4:**
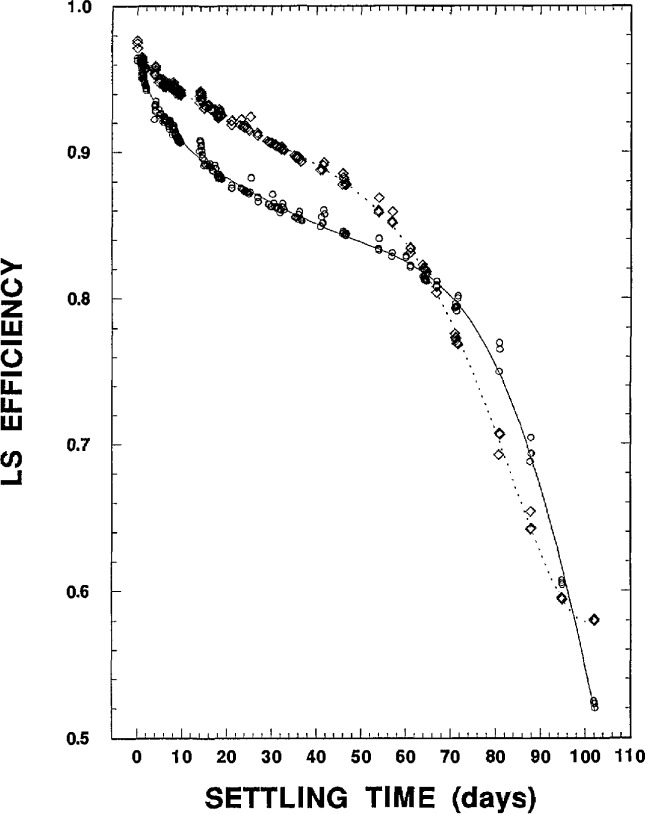
^36^CI LS efficiency data for the two precipitated samples for settling times up to 102 d. The dotted curve is that for the 219 mg NaCl sample; the solid curve for the 757 mg NaCl sample. Refer to the text for discussion.

After settling times greater than about 60 d, however, the efficiency data for both the 219 mg and 757 mg NaCl sources (see Fig. 4) had abrupt and very rapid decreases. The less massive 219 mg source began to change at around 65 d to 70 d, and its efficiency dropped to nearly 60% within the next 30 d; whereas the more massive 757 mg source did not exhibit this change until about 75 d to 80 d, but then underwent an even more rapid efficiency drop to less than 55% within the next 20 d. This unexpected and inexplicable behavior precluded hopes of ever finding a steady-state efficiency condition for counting the precipitated-sample sources.

The quite noticeable wide fluctuations and variations in the efficiency versus settling time data (Figs. 2–4) were also disconcerting and disappointing. The cause of this substantial irreproducibility was initially unknown. Replicate and sequential measurements on the same sample often exhibited greater efficiency variations than efficiency values obtained at much later settling times. These variations could be as great as several percent. Similarly, the measured efficiencies on a given sample could on occasion even exhibit increases as a function of increasing settling time. This irreproducibility was eventually attributed to partial stirrings of the settled precipitates when the counting sources moved up and down on the elevator of the automatic sample changer for the LS counting system.

Therefore, the best one might hope for in attempting to assay solution D was to try to make relative measurements against solution A for closely matched precipitated samples (i.e., in terms of their total NaCl loading) and as a function of matched settling times.

**Fig. 5:**
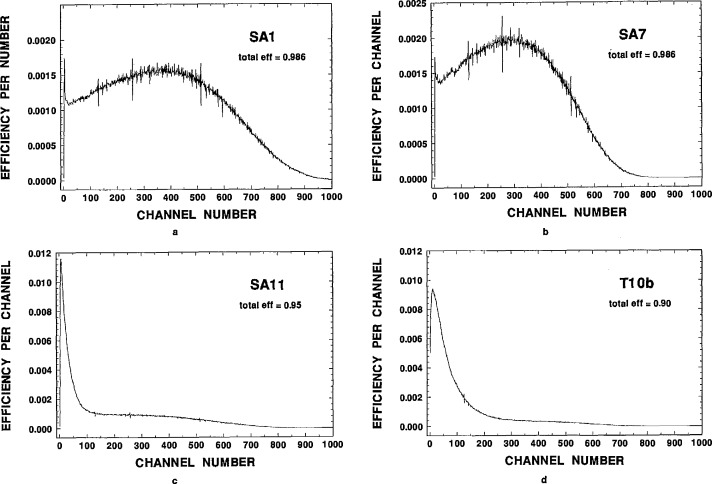
Typical ^36^Cl LS spectra of cocktails loaded with varying NaCl content: (a) SA1, a clear sample containing ⪝0.02 mg NaCl in 0.22% water; (b) SA7, a clear sample containing 111 mg NaCl in 93% water; (c) SAH, a precipitated sample containing 227 mg NaCl in 9.6% water; and (d) Tl0b, a precipitated sample containing 757 mg NaCl in 26% water. Refer to the text and tables for typical quench parameters and efficiencies under these conditions.

Comparison of the ^36^C1 LS spectra for different NaCl loadings in both clear and precipitated-sample sources are also informative. Figure 5 contains four typical spectra under four widely differing conditions. The spectra, obtained with the Packard LS system, are presented in terms of the measured efficiency per channel for samples containing known quantities of ^36^C1 as a function of the channel number. The channel numbers are directly proportional to *β^−^* energies. The first spectrum, for sample SA1 (see Table 3), is that with least quenching. This sample was a clear solution containing ⪝0.02 mg NaCl and about 0.22% water, and had an efficiency of 0.986. The spectral shape has the overall, general appearance of that expected for an undistorted ^36^C1 beta-ray spectrum (having a single, non-unique second forbidden *β*^−^-transition) [12]. The second spectrum, for sample SA7, is only modestly quenched from that of first, although the sample contains 109 mg NaCl and 9.9% water. The total efficiency, summed over all channels for this sample, is still 0.986, but the spectral shifts to lower energies are quite evident. Other spectra for samples of composition intermediate between the above two also exhibit these very gradual shifts between the extremes of the above two spectra. With any further increases in the NaCl content in the samples to the point at which precipitates begin to form, the spectral shapes change dramatically. The spectrum for precipitated sample SAH (containing 227 mg NaCl and 9.6% water) in Fig. 5 shows this dramatic change. The doubling of the NaCl content in this sample from that of the last spectrum resulted in a drastically compressed spectral shape. The total efficiency was still quite large at about 95%, but a substantial fraction of this efficiency derived from detected events at energies corresponding to channel numbers of less than 100. The final spectrum in Fig. 5 for sample Tl0b (see Table 8) shows the continuing spectral shifts to lower energies and to lower total efficiencies with increasing NaCl content in precipitated samples. Other spectra (not shown) for precipitated samples as a function of settling time exhibit identical effects.

#### 2.1.4 LS Counting of Solution D

Two LS counting sources containing gravimetrically-determined aliquots of solution D were prepared to conduct the assay. The samples, labelled Dl and D2, contained 5.2075 g and 5.5432 g of solution D in 15.160 g and 15.439 g of scintillator, respectively. Both samples contained massive precipitates; and their total loadings corresponded to 755 mg NaCl and 34.4% water for D1, and 804 mg NaCl and 35.9% water for D2

The samples were measured relative to matched precipitated samples of solution A, and with corresponding matched blanks, on the Beckman LS system. The samples were matched in terms of total NaCl mass and the approximate water content (mass percent) in each cocktail. Each experimental trial consisted of 8 to 15 replicate measurement cycles. Each cycle consisted of sequential measurements of a solution D sample (either Dl or D2), a matched blank for background subtraction, and matched solution A samples. The three sample types (D, blank, and A) were measured alternately in order to closely match settling times between samples. Counting times for the measurement of each sample ranged from 6,000 s to 18,000 s. The total counting time on each sample for a given trial (i.e., 8 cycles to 15 cycles) therefore ranged from 60,000 s to 180,000 s. Such long counting time intervals were required because of the low total counting rates for the solution D samples.

Five such closely matched trials on each solution D sample (Dl and D2) were performed, and the results are summarized in Table 9. For each trial, the table provides the mean count rate concentration (cps · g^−1^) obtained by averaging the individual background-corrected count rates obtained from the *n* measurement cycles, the calculated relative standard deviation of the mean *s*_m_ for the measurement set, and the total statistical (Poisson) counting precision, in terms of a relative standard deviation *s*_p_, calculated from the total number of sample and background counts obtained in the *n* measurement cycles. As indicated, the magnitude of both the *s*_m_ and *s*_p_ precision estimators are substantial, typically ranging from 2% to 4%. This appreciable imprecision is due in part to the large measurement variations obtained with precipitated samples as discussed previously in Sec. 2.1.3, and in part to the large statistical “counting error” for these low level samples. The net sample count rate was typically only about 15% of background. For example, the first row entry for sample Dl in Table 9 had an average background count rate of 1.071 cps (*S*_m_=0.59%), gross counting rates ranging from 1.196 cps to 1.263 cps, and hence net rates of about 0.125 cps to 0.192 cps for the sample (with a mean of 0.157 cps and *s*_m_ = 4.4%).

The assumed efficiencies in Table 9 were obtained from averaging the LS results for the solution A samples across all matched measurements within an *n* -measurement cycle trial. The ^36^C1 activity concentration in samples Dl and D2 were then calculated from the similarly averaged Dl and D2 mean count rate concentrations and these efficiency values. The data were also analyzed by calculating the individual D to A ratio for each adjacent measurement pair within a cycle, and then averaging the resulting *n* measurement ^36^C1 activity concentrations. The results of the two calculational approaches were invariant within statistical precision. The uncertainties in the efficiencies determined in this way were estimated to be ±2% to ± 3% at an assumed one standard deviation uncertainty interval. This estimation may be better appreciated by examination of the settling data of Fig. 1. The vertical dotted lines at around 750 mg and 800 mg NaCl correspond to the NaCl content in samples Dl and D2. The three dotted horizontal lines in Fig. 1(a) represent best estimates of the mean efficiency (0.88), and the lower and upper bounds on the uncertainty interval which corresponds to 0.88 ±0.025. Estimates of the assumed efficiency means are similarly illustrated in Figs. 1(b) and 1(c). These uncertainty estimates however do not directly address possible additional uncertainty components due to sample composition mismatching differences.

It should also be mentioned that the timings of the matched measurement trials (data of Table 9) were in part selected to correspond to settling times having a fairly systematic regularity, such as the near linear region exhibited in Fig. 3.

The last column of Table 9 compares the ten independently-determined values of the solution D activity concentration to that reported for the AMS standard (compare Table 1). These activity concentration ratios are also equivalent, however, to ratios of the reported to measured D/A dilution factors. Table 10 summarizes these comparison ratios in terms of several computed statistical estimators. It is evident that there are no substantial differences between the grand means for sample Dl, D2, and both samples. Because of this, as well as the large number of replications and sampling combinations that comprise these grand means, it is reasonable to conclude that the additional uncertainties due to possible sample composition mismatches for these precipitated samples were not dominant, and that they did not introduce a significant bias error. There are also no significant differences in the three averaging methods used to calculate the grand means. These summarized results (Table 10) clearly confirm the solution D activity concentration relative to that of solution A (or equivalently the D/A dilution factor) to an absolute difference of roughly 1%, and to better than 2% in worst cases. The uncertainty in these comparisons, mainly on consideration of the values of *s*_m_ and *s*_P_, are roughly ±3% at a relative three standard deviation uncertainty interval. All other possible contributing uncertainty components, e.g., those due to radioactive decay corrections, background subtraction corrections, dead time counting corrections, timing measurements, gravimetric determinations, LS cocktail stability, or efficiency mismatches arising from sample composition and quenching differences are either embodied in the overall uncertainty estimate or are negligible.

### 2.2 Proportional Counting Measurements

An additional sequence of assay measurements of solutions A, B, C, and D were performed by low-background 2π*β*^−^ gas-flow proportional counting of solid sources. It was known *a priori* that their results were likely to be less accurate than those obtained by LS counting; and therefore, they were only intended to serve as secondary, confirmatory measurements. In addition, these solid source measurements were in large part designed to serve as an experimental backup for the assay of solution D, and were initiated before it was known whether LS counting of solution D was feasible.

The counting sources were prepared by dispensing gravimetrically-determined aliquots of the solutions and, in some cases, appropriate quantities of the NaCl blank solution onto source mounts which consisted of circular disks of 39 mm diameter ion exchange papers that were fixed onto larger 52 mm diameter stainless steel planchet inserts. The dispensed solutions were subsequently evaporated to dryness in air, and the mounts were then covered and sealed with a mylar film having a surface density of 1.75 mg · cm^−2^. The ion exchange paper used for the source mounts was a Reeve Angel Amber-lite, grade SB-2, filter matrix containing IRA-400 resin in Cl^−^ form which is a strong-base-type anion exchanger.

Two sources for each solution were prepared as summarized in Table 11. For solutions A and B, the order of dispensing the active and blank solutions was reversed in preparing each of their two sources. For the first sources (either Al or Bl), the blank solution was dispensed first and followed by the addition of the active (either A or B) solution; whereas for the second source (either A2 or B2), the active solution was dispensed first and followed by the blank solution. They were prepared in this way so that any possible differences in the *β^−^* self absorption due to the addition of the blank solution could be accounted for. The sources for solutions C and D were prepared with undiluted aliquots of the solutions.

The evaporated sources were visibly irregular in the distribution and thickness of the NaCl crystals. The average NaCl-crystal surface densities for the sources ranged from about 10 mg · cm^−2^ to 13 mg · cm^−2^. Although undesirable from a *β*^−^ counting perspective, the use of such thick self-absorbing sources was necessitated by the low activity concentration for solution D that required the use of large sample aliquots. In order to make relative measurements, the sources for solutions A, B, and C were matched to those for solution D.

The 2π*β*^−^ measurements of the solid sources were performed with a Berthold LB-770 11-channel low-level planchet counting system. The system comprises ten separate gas-flow proportional counter tubes that allow the simultaneous measurement of ten 60 mm diameter sample planchets, and a common guard counter tube, located above the ten measuring counter tubes, that acts as an anticoincidence shield to achieve low backgrounds. The counting gas was a “P-10” argon-methane (90:10) mixture. The counters are housed in a 10 cm lead shield for additional external radiation shielding.

Typical background count rates for the ten counters range from about 0.2 to 0.9 counts per minute when operated on the high voltage beta plateau. In comparison, the large flat guard counter has a background rate of about 650 counts per minute. Counter tubes located at the ends of the housing have the larger backgrounds because of less lead and anticoincidence shielding. Therefore, the center or middle counters were chosen for measurement of the lower level sources.

The background considerations were critically important. Assuming even an idealized 2π detection efficiency of 0.5 with no self-absorption losses, a 0.03 Bq solution D source would provide a net count rate of only about 0.9 cpm. Hence, the background count rates for even the lowest-background counters (i.e., 0.2 cpm) made substantial contributions to the gross count rates for the solution D sources and needed to be determined very precisely.

The crossover of stray and scattered radiation to the counters from adjacent source positions was reported by the manufacturer to be approximately 0.002% for a pure beta (^90^Sr-^90^Y) source and less than 0.1% for a beta-gamma (^137^Cs) source. Somewhat larger stray factors were observed with the ^36^C1 (a nearly pure beta emitter) solution A sources used in this work. The largest factor, for one of the end counter tubes with an adjacent source, was 0.3%. The factors for middle or center counters, again with adjacent sources, were nearly an order of magnitude less.

**Fig. 6:**
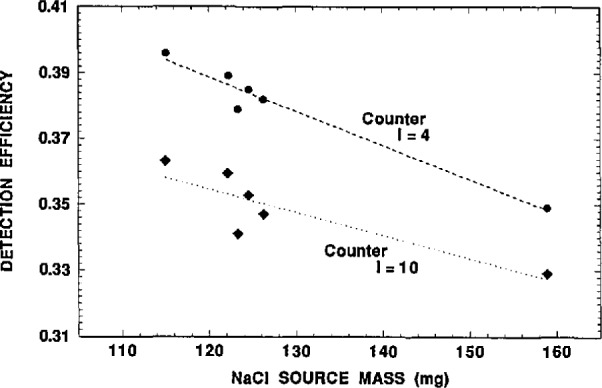
Apparent 2π*β*^−^ gas-flow proportional counting detection efficiency for two counters obtained with the solid sources of solutions A, B, and C assuming the reported (Table 1) ^36^C1 activity concentrations.

Detection efficiency variations between the ten counters were too large to rely on the use of an average efficiency. The observed efficiency variations for ^36^C1 between the six lowest-background counters ranged from 1% to 6% based on measurements of one source across the counters. The apparent detection efficiencies for the sources were in the range of 0.33 to 0.40. Figure 6 illustrates the observed efficiencies, and the *β^−^* self absorption in the solid sources for the six sources of solutions A, B, and C as measured in two of the counters. The reported activity concentrations for solutions A, B, and C (Table 1) were assumed in deriving these apparent efficiencies. Excepting the most massive source (A2), the variations in efficiency (within a given counter) for the other more closely matched sources were, of course, much smaller. The apparent efficiency variations between these other five sources, mainly due to differences in the source *β^−^* self absorption, were about ±2% to ±3% as indicated in the data of Fig. 6. These observed dispersions however also include contributions from the statistical counting imprecision and differences arising from source positioning and placement in the counters.

Dead-time count rate losses were fairly negligible for any of the sources. The electronic pulse resolving times for the proportional counters were of the order of τ = 10 μs, thereby giving dead-time corrections ranging from less than 0.2% for the solution A sources, to less than 0.02% for the solution B sources, to less than 0.001% for the solution C sources, and less than 0.00001% for the solution D sources.

With the above source characteristic, background, detection efficiency, and *β^−^* counting considerations in mind, the experimental design for the measurements was based on the following criteria: (1) a relative measurement between any two sources required that both sources be counted on the same counter to assure equivalent detection efficiencies; (2) the background count rate for that same counter needed to be precisely determined; (3) measurement of several different combinations of the sources for each solution pair were required to account for possible differences in source preparation and *β^−^* self absorption in the sources; (4) the relative determinations for the various solution pairs also needed to be based on measurements in several counters; (5) measurements of different sources and backgrounds on the same counter needed to be interspersed to account for any temporal differences; (6) long counting time intervals, for the backgrounds and low count rate sources, were required to achieve good statistical precision; and (7) a large number of replicate measurements were needed to account for possible differences in positioning the sources in the counters.

**Fig. 7:**
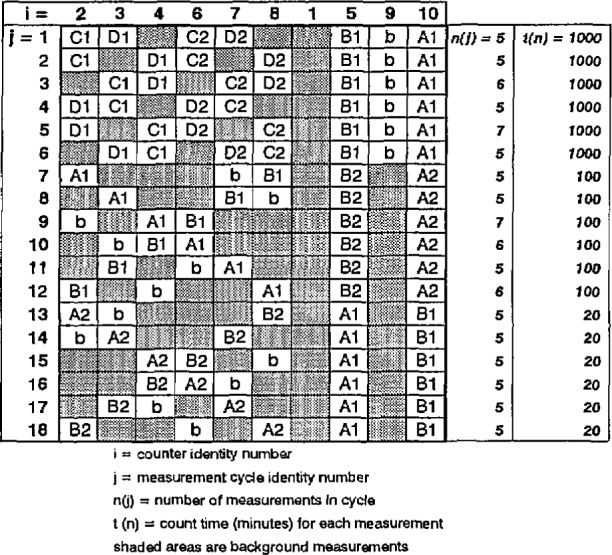
Experimental design for the 2π*β^−^* gas-flow proportional counting measurements of evaporated solid sources of solutions A, B, C, and D. The design consists of a matrix of the location and measurement sequence of the counting sources in chamber *i* for measurement cycle *j* with *n_j_* replicate measurements, each of counting time duration *t_n_*.

The experimental design employed for the measurements is outlined in the matrix of Fig. 7. The matrix provides the locations of the sources alternately counted in the various counters as a function of the measurement sequence. Each measurement cycle *j* consisted of five to seven replicate measurements where each replicate was of 20 min to 1000 min in duration. Sources for solutions C and D (C1, C2, Dl, and D2) were measured interchangeably, as shown, in the lowest-background counters *i* = 2, 3, 4, 6, 7, and 8. The shaded elements of the matrix represent background measurements of equal time duration. Counters 5, 9, and 10 were used as controls with solution A and B sources in case any normalizations between measurement cycles were necessary. Counter 1 was used as a background control for all measurement cycles. Measurement of the blank source (b) was interspersed between counters and measurement cycles. The total continuous counting time for all 18 measurement cycles exceeded 25 d of live time (37,000 min) which was conducted over approximately 38 d of real time.

**Fig. 8:**
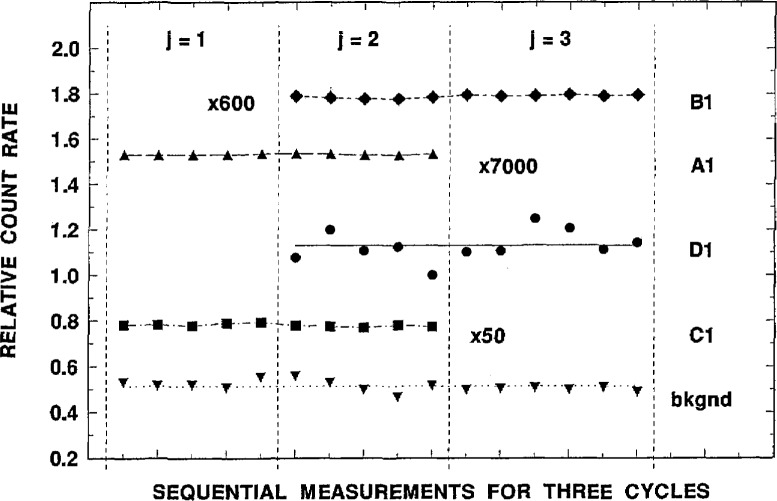
Typical 2π*β^−^* gas-flow proportional counting measurement results in terms of relative count rates for sequential measurements over three measurement cycles *j* for five sources: B1 (measured in counter *i* =5); A1 (*i* = 10); Dl (*i* =4); C1 (*i* =2); and background (*i* = 1).

The results of these counting measurements consisted of a huge [18 cycle × 10 counter × 5 to 7 measurement] three-dimensional matrix of data which was reduced to derive a substantial number of relative determinations of the activity concentrations, first for the various source pairs, and ultimately for their respective solution pairs. For example, the results from any one of the counters *j* =2, 3, and 4 provide between 10 to 12 independent measurements of C1, Dl, and the background (cycles *j* = 1 through 6) as well as five to seven measurements on each of sources Al, A2, Bl, and B2 along with an additional two measurements of blank source b and eight measurements of background (cycles 7 through 18). These results not only provide average determinations of the Cl/Dl source activity concentration ratio for each of the three counters, but also determinations of the Bl/Dl, B2/D1, Al/Dl, A2/Dl, Bl/Cl, B2/C1, Al/Cl, A2/C1, Al/Bl, A1/B2, A2/B1, and A2/B2 ratios for each counter. Similarly, the results from any one of the *j* = 6, 7, and 8 counters provide equally determined ratios for the C2/D2 source pair, and another comparable set of determinations for all the same source pairs listed above. Table 12 summarizes the total number of determinations that were made for all possible combinations of the various source pairs on each counter to provide their respective solution pair ratios. In examining Table 12 it is important to note that, based on the experimental design (Fig. 7), not all source pair ratios for a given counter were determined with equal numbers of measurements for both sources. Table 13 summarizes the total number of measurements made in each counter for each source. In analyzing the data set, the measurement results for a given source pair combination (within a given counter *i*) was averaged over all appropriate measurement cycles *j*. Analyses of smaller subsets of the data in terms of calculating the ratios for individual *i* and *j* combinations indicated that there were no significant differences with the results obtained by averaging over all cycles. Obviously, this conclusion would equally be valid as long as there were no significant differences in the source count rates between cycles within a given counter. Figure 8 demonstrates the good reproducibility in the relative count rates for four sources and background over three measurement cycles, *j* = 1, 2, and 3. The plotted results for three of the sources (Bl, Al, and C1) in Fig. 8 were normalized by somewhat arbitrary factors merely to display the results on a similar scale. These illustrated data, obtained over an approximate 11-day interval, are representative and typical.

Detailed results for the determined C/D solution pair ratios are provided in Table 14. These results are based on the Cl/Dl (in counters *i* =2, 3, and 4) and C2/D2 (in counters *i* = 6, 7, and 8) source pairs from the first six measurement cycles (*j* = 1 through 6). The results are tabulated in terms of the mean count rate for each source in each detector averaged over all the *n* = 10 to 12 measurements in both counting cycles, and the two precision estimators, *s*_m_ and *s*_p_, for each mean. The magnitudes of *s*_m_ and *s*_p_ clearly indicate the much better precision in the measurement of the solution C sources compared to the solution D or background measurements. The activity concentration ratios (last column of Table 14) were then obtained from the relation

$$R_{C/D}=\frac{(d_{C}-d_{\text{bkgnd}})/m_{C}}{(d_{D}-d_{\text{bkgnd}})/m_{D}}$$

where *d*_C_ and *d*_D_ are the mean count rates for the solution C and D sources, *d*_bkgnd_ is the mean background count rate, and *m*_C_ and *m*_D_ are the aliquot masses of the C and D solution sources. No dead-time loss corrections were made, nor were any attempts made to try to account for differences in *β^−^* self absorption in the sources. The six determinations (three for the Cl/Dl source pair, and three for C2/D2) have a mean ratio of 52.37 with a standard deviation of the mean of *s_m_*(*n* =6) =2.0%. There are no significant differences between this mean ratio and the mean obtained from just the Cl/Dl source pair values (52.08) or that for the C2/D2 pair values (52.65). Analyses of the mean ratio results across other variables such as by counter number *i* for a specific source pair and cycle number *j*, based on t-tests of differences in the various means and on χ^2^- and F-tests of the homogeneity in the various subset sample means and variances, indicated that there were no statistically significant differences in any of the tested subset sample means or variances.

Similar analyses were performed on all the other 76 source pair combinations (see Table 12) to obtain the mean solution pair ratios. For the sake of brevity, the detailed counting result data (analogous to that presented in Table 14) for these are omitted here. A summary of the cumulated mean ratios for all of these determinations are however tabulated in Table 15, which also contains the comparisons to the reported ratios of Table 1. The first row of Table 15, for example, devolves directly from the analyzed results of Table 14. As before, subsets of the mean ratio results across the source, counter number, and measurement cycle variables were tested (t-, *χ*^2^-, and *F*-tests, as well as by sequential 2-variable ANOVA techniques) for differences in subset means and variances. Excepting a difference in ratios obtained with sources Al and A2, attributed to *β*^−^ self absorption differences (see below) in these two sources, all of the other subset sample means and variances were statistically equivalent.

The mean activity concentration ratios or dilution factors for the solution pairs have relative standard deviations of the mean *s*_m_ ranging from less than 0.2% for the 28 determinations of the A/B ratio to over 4% for the A/D ratio. As somewhat expected, the *s*_m_ values are typically smaller for those solution pairs that are closer in activity concentration (A/B, B/C, and C/D), and increasingly larger for those having greater activity concentration differences (A/C, B/D, and A/D). The very small *s*_m_ = 0.17% value for the A/B ratio was also surprising in that there was a statistically significant difference in the A1/A2 source pair ratios across all *i* and ; counter number and measurement cycle variables. The difference was attributed to the difference in *β^−^* self absorption in the more massive A2 source. The average A1/A2 difference observed over all measurements in all seven counters was 4.6%. No attempt was made however to account for this observed absorption difference in the A2 and Al sources in deriving any of the solution A ratios. That is, the ratios with solution A were derived by averaging the mean count rate concentration ratios over the averages of both the A2 and Al sources. As stated above, there were no similarly observed statistically significant differences in the results for any of the other solution B, C, or D sources. The invariance in the Cl/Dl and C2/D2 means of Table 14, discussed previously, is illustrative.

The comparisons between the reported activity concentration ratios or dilution factors *R* of Table 1 and the measured ratio *R*_m_ ranged from *R/R*_m_ = 1.040 for A/B to *R/R*_m_ = 1.180 for A/D. Again, it is perhaps not surprising that the greater differences were obtained for solutions having greater concentration differences (A/C, B/D, and A/D) and less for those having smaller concentration differences.

**Fig. 9:**
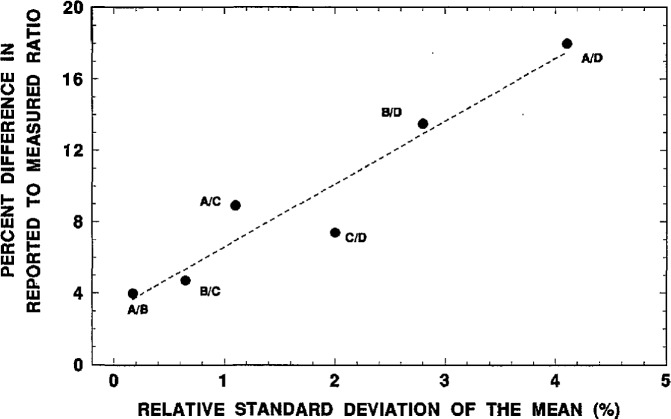
Correlation between the absolute percent difference in the reported to measured ratios for the various solution pairs and the relative standard deviation of the mean for the measured ratio obtained by 2π*β*^−^ gas-flow proportional counting of evaporated solid sources.

One of the more surprising, and rather inexplicable, findings is that all of the tabulated solution pair ratios are positively biased, i.e., *R/R*_m_>1. For every tabulated solution pair, the measurement result for the higher activity concentration solution source is always in the denominator of the comparator *R/R*_m_. This might suggest the existence of some type of measurement bias that is systematic with increasing activity concentration, e.g., dead-time losses. The large magnitudes of the effect however would seem to exclude this as a possibility. Another interesting finding was that the differences between *R* and *R*_m_ were strongly correlated with the measurement precision. Figure 9 shows the correlation plot for the *R/R*_m_ difference as a function of the relative standard deviation of the mean *s*_m_. The reduced correlation coefficient is 0.957. This does, in part, imply that the magnitudes in the observed differences between the reported ratios *R* and the measured values *R*_m_ are indeed dependent on the measurement precision. The analyses of the overall uncertainties on these measurements (Table 16) further indicate that the magnitudes of the *R/R*_m_ differences can largely be attributed to the measurement uncertainties. Nonetheless, these uncertainty arguments somewhat beg the question of why the apparent differences in *R/R*_m_ were all positively biased in all six solution pair cases (Table 15). Any reasonable explanation for this remains unknown.

In conclusion, these 2π*β*^−^ gas-flow proportional counting measurements, intended to be secondary, confirmations of the LS results, verified the reported dilution factors to about 4% to 8% for the single-dilution solution pairs (A/B, B/C, and C/D) and to roughly 9% to 18% for the double- and triple-dilutions (A/C, B/D, and A/D solution pairs).

## 3. Summary and Concluding Notes

The first three dilutions in the eight-step serial gravimetric dilution scheme that was used to prepare a series of ^36^C1/C1 isotopic ratio AMS standards were verified by the relative radioactivity measurements on four ^36^C1 solutions described hereinbefore. The results, based on liquid scintillation counting of sample aliquots of the diluting solutions along with confirmatory measurements by gas-flow proportional counting of evaporated solid sources, were treated and discussed *in extenso* and are principally summarized in Tables 6, 10, and 15. In all cases, the reported gravimetric dilution factors were verified to absolute differences of better than 2%.

Beyond the findings of these validation measurements however, the investigations described here were also significant in terms of broadening the applications of several conventional techniques in radionuclidc metrology.

The assay of *β^−^* emitting radionuclides by 4π*β^−^* LS counting, particularly for reasonably energetic nearly pure beta emitters like ^36^C1, was previously well known, well documented, and somewhat straightforward. These LS techniques however were mainly employed with homogeneous solution cocktails having only moderate sample quenching. This study demonstrated the potential for LS counting of samples quenched with large quantities of NaCl. Even for samples loaded with in excess of 100 mg NaCl, the cocktails appeared sufficiently stable, and the LS efficiencies remained surprisingly large at greater than 98%.

The discovery of the feasibility of performing highly-accurate LS measurements of precipitated samples was even more significant. This type of assay procedure is of necessity more complex and demanding. It requires relative measurements of closely matched precipitated samples as a function of matched settling times. Although the measurement precision with these very heterogeneous samples is inherently worse compared to conventional LS counting, it is possible with sufficient measurement trials and replications to achieve overall measurement uncertainties of a few percent.

Similarly, the results of the 2π*β*^−^ gas-flow proportional counting measurements clearly demonstrated the potentialities and power of a proficient experimental design even when dealing with an intrinsically inferior measurement method. The large *β*^−^ self-absorption losses in the rather thick solid sources and other attendant variabilities in the proportional counting still did not exclude the possibility of performing reasonably accurate assays.

It may be useful, in conclusion, to reconsider the difficult nature of these radioactivity measurements. They required solution assays that were nearly beyond the capability of available methodologies and technology. The solutions contained 145 mg NaCl per gram of solution and had ^36^Cl activity concentrations ranging from 11 kBq · g^−1^ to 0.036 Bq · g^−1^, the latter having net counting rates that were only a few percent of typical backgrounds. Yet, the assays were performed with overall measurement uncertainties, corresponding to a relative three standard deviation uncertainty interval, of ± 1% to ±3% in general for the LS counting measurements and about ±5% in best cases for the gas-flow proportional counting measurements. If anything then, this work illustrated that even when confronted with a difficult, seemingly impossible measurement task [such as that resulting from the worst conflicting combination of a sample containing a large carrier (e.g., salt) mass and a very low activity concentration], it is often possible to adapt, modify, or extend conventional methodologies to that task. This requires, of course, careful experimental designs and plans, painstaking and exacting metrological practices, and, perhaps most importantly, dogged determination and tenacious perseverance. This paper is no doubt a surprise to those individuals who believed that the assay of solution D by radioactivity measurements was not doable.

## Figures and Tables

**Table 1:** Reported compositions of the serial dilution solutions used to prepare the ^36^Cl/Cl AMS standards

Solution identity	Isotopic ratio ^36^C1/C1	NaCl concentration (mg · g^−1^)	^36^Cl activity concentration (Bq · g^−1^)	Gravimetric dilution factors for ^36^C1
A^a^	7 (10^−2^)^b^	0.02^b^	10950.	
B	1.059 (10^−6^)	144.0	114.6	A/B = 95.55
C	1.825 (10^−8^)	145.5	1.997	B/C = 57.39
				A/C = 5483.
D	3.245(10^−10^)	145.6	0.03551	C/D = 56.24
				B/D = 3227.
				A/D = 308400.

a NIST SRM 4943.

b Nominal values.

**Table 2:** Expeeted ^36^CI LS efficiency for unquenched samples

		Assumed ^36^Cl 4π*β*^−^ LS effieieney^a^		
		1.000	0.995	0.990
Assumed ^36^Cl	0.75	0.995	0.990	0.985^c^
4π EC LS	0.50	0.991	0.986^c^	0.981
Efficiency^b^	0.25	0.986^c^	0.981	0.976

a For 98.1% *β^−^* (maximum *E_β_−* =709.3 keV; average *E_β_−* = 251.2 keV).

b For 1.9% EC.

c Observed in least quenehed samples; *ϵ*_lot_ = 0.985 to 0.987.

**Table 3:** LS counting efficiency for ^36^Cl as a function of NaCl sample loading

Sample identity	LS Sample composition^a^				LS Counting results		
	Aqueous mass percent^b^	NaCl mass (mg)	^36^C1activity(Bq)	Appearance	Average	Efficiency	*s*_m_ (%)^d^
SA1	0.22	(e)	355.8	clear	64	0.9870	0.047
SA2	0.29	(e)	477.9	clear	65	0.9850	0.027
SA3	9.09	(e)	223.2	clear	94	0.9856	0.084
SA4	9.41	(e)	715.3	clear	95	0.9776	0.039
SA5	9.86	44.59	354.8	clear	96	0.9871	0.067
SA6	10.5	49.58	561.8	clear	97	0.9846	0.039
SA7	9.58	109.0	362.5	clear	94	0.9845	0.049
SA8	9.15	143.3	467.9	ppt. (trace)	85	0.9833	0.079
SA9	9.26	146.5	447.6	ppt. (trace)	85	0.9813	0.059
SA10	9.13	212.6	521.7	ppt.	72	0.9311	0.037
SA11	9.57	227.0	303.9	ppt.	72	0.9507	0.15

a In approximately 15.06 g scintillator fluid.

b [*m*_water_/(*m*_water_ + *m*_scin_)] 100.

c Horrocks number (see text).

d Relative standard deviation of the mean in percent for four measurements on each sample. The total statistical (Poisson) counting precision in terms of a relative standard deviation was about 0.05% for each sample.

e Minimal; approximately ⩽0.01 mg NaCl.

**Table 4:** LS results for solutions A, B, C obtained with Beckman LS 7800 counter

Solution	Sample identity	LS Sample composition^a^				LS Counting results for ^36^C1			
		Aqueous mass percent^b^	NaCl mass (mg)	Mean rate (cps · g^−1^)	*s*_m_^c^ (%)	*s*_p_^d^ (%)	Average *H* #^c^	Efficiency	Activity concentration (Bq · g^−1^)
A	SAl^f^	0.22	g	10800	0.038	0.036	60	0.9863	(10950)^h^
	SA2^f^	0.29	g	10788	0.020	0.031	61	0.9852	(10950)
	SA7^f^	9.58	109.	10799	0.032	0.035	92	0.9862	(10950)
	Al	9.29	111.	10800	0.037	0.036	94	0.9863	(10950)
	A2	9.43	109.	10770	0.012	0.031	95	0.9836	(10950)
	A3	9.55	113.	10774	0.035	0.031	95	0.9839	(10950)
B	Bl	8.96	105.	113.09	0.15	0.13	94	(0.985)^h^	115.6
	B2	9.12	110.	113.23	0.11	0.090	93	(0.985)	115.0
	B3	8.90	104.	113.07	0.052	0.075	91	(0.985)	114.8
C	C1	9.12	110.	1.9399	0.524	0.85	91	(0.985)	1.969
	C2	9.11	110.	1.9551	0.463	0.85	91	(0.985)	1.985
	C3	9.04	108.	1.9607	1.639	0.86	91	(0.985)	1.991

a In approximately 15.05 g scintillator fluid.

b [*m*_water_/(*m*_water_ + *m*_scin_)] 100.

c Relative standard deviation of the mean in percent for five measurements on each sample.

d Relative standard deviation in percent for the total statistical (Poisson) counting precision.

e Horrocks number (see text).

f Previous samples of Table 2.

g Minimal; approximately ≤ 0.01 mg NaCl.

h Values in parentheses are cither the known activity concentration for solution A used to calculate the counting efficiencies or arc the assumed efficiencies used to calculate the activity concentrations for solutions B and C.

**Table 5:** LS counting results for solutions A, B, C obtained with Packard 2500 TR counter

Solution	Sample identity^a^	LS Counting results for ^36^C1					
		Mean rate (cps · g^−1^)	*s*_m_(%)^b^	*s*_p_(%)^c^	Average *tSIE*	Efficiency	Activity concentration (Bq · g^−1^)
A	SA1	10810	0.040	0.036	578	0.9872	(10950)
	SA2	10810	0.041	0.031	571	0.9872	(10950)
	SA7	10803	0.038	0.035	439	0.9866	(10950)
	Al	10825	0.031	0.036	437	0.9886	(10950)
	A2	10789	0.035	0.031	436	0.9853	(10950)
	A3	10802	0.025	0.031	431	0.9865	(10950)
B	Bl	113.06	0.13	0.13	440	(0.987)^e^	114.5
	B2	113.34	0.092	0.089	439	(0.987)	114.8
	B3	113.33	0.082	0.075	444	(0.987)	114.8
C	C1	1.9382	0.56	0.82	441	(0.987)	1.964
	C2	1.9487	0.52	0.82	439	(0.987)	1.974
	C3	1.9611	0.38	0.82	441	(0.987)	1.987

a See Table 3 for LS sample composition.

b Relative standard deviation of the mean in percent for five measurements on each sample.

c Relative standard deviation in percent for the total statistical (Poisson) counting precision.

d Transformed spectral index of the external standard (see text).

e Values in parentheses are cither the known activity concentration for solution A used to calculate the counting efficiencies or are the assumed efficiencies used to calculate the activity concentrations for solutions B and C.

**Table 6:** Comparison of reported to measured dilution factors for solutions A, B, and C

Solution pairs	Ratios of averaged LS results			Reported gravimetric dilution factor (*R*)^c^	*R/R*_LS_
	With Beckman counter^a^	With Packard counter^b^	Both Counters (R_LS_)		
A/B	95.37	95.43	95.39	95.55	1.002
A/C	5527.	5544.	5535.	5483.	0.991
B/C	57.96	58.09	58.03	57.39	0.989

a See Table 4.

b See Table 5.

c See Table 1.

**Table 7:** Uncertainty analysis for the measured dilution factors for solutions A, B, and C

Solution pair	LS counter	LS Measurement precision (%)^a^	Combined uncertainty, *u* (%)^b^	Overall uncertainty, 3*u* (%)^c^
A/B	Beckman	0.062	0.33	1.0
	Packard	0.058	0.33	1.0
B/C	Beckman	0.56	0.64	1.9
	Packard	0.27	0.41	1.2
A/C	Beckman	0.55	0.64	1.9
	Packard	0.26	0.41	1.2

a Corresponds to the combined relative standard deviation of the mean in percent for the precision in the LS counting rate concentration ratios. See Tables 4 and 5.

b Includes uncertainties due to the LS measurements (0.05% to 0.1%), and differences or mismatch in LS efficiency due to differences in sample composition, quenching, and cocktail stability.

c Corresponds to three times the combined uncertainty *u* which is assumed to provide an uncertainty interval having a high level of confidence of roughly 95% to 99%.

**Table 8:** LS counting efficiency for ^36^Cl in precipitated samples.

Sample identity	LS Sample composition^a^			LS Counting results					
	Aqueous^b^ mass percent	NaCl mass (mg)	^36^Cl activity (Bq)	First trial^c^			Second trial^d^		
				Mean efficiency	*s*_m_^e^(%)	*H#*^f^	Mean efficiency	*s*_m_^e^(%)	*H#*^f^
T1	5.20	109.	901.6	0.953	0.14	75	0.964	0.14	62
T2	9.61	219.	1141.	0.949	0.086	78	0.953	0.11	65
T3	14.7	363.	1220.	0.929	0.14	78	0.951	0.046	68
T4	17.0	436.	1117.	0.923	0.068	77	0.946	0.062	68
T5	19.4	514.	1084.	0.916	0.12	78	0.935	0.10	70
T6	21.3	583.	874.8	0.900	0.031	80	0.931	0.070	71
T7	23.3	656.	1107.	0.895	0.097	80	0.926	0.060	73
T8a	24.1	684.	1024.	0.878	0.14	81	0.922	0.070	74
T8b	24.1	687.	909.0	0.889	0.16	82	0.922	0.16	75
T9a	25.2	729.	914.5	0.890	0.083	81	0.918	0.037	76
T9b	25.2	728.	947.9	0.860	0.10	85	0.924	0.088	76
T10a	25.9	757.	941.7	0.877	0.090	86	0.919	0.094	78
T10b	25.9	757.	868.6	0.906	0.11	82	0.921	0.15	80
T11	27.0	800.	970.2	0.878	0.12	87	0.916	0.070	81

a In approximately 15.17 g scintillator fluid. All samples had visible precipitates in the cocktail.

b [*m*_water_/(*m*_water_ + *m*_scin_)] 100.

c Obtained after 2 to 4 d of settling. Refer to text.

d Obtained after the samples were centrifuged. Refer to text.

e Relative standard deviation of the mean in percent for five measurements on each sample. The total statistical (Poisson) counting precision in terms of a relative standard deviation was less than 0.01% for each sample.

f Average Horroeks number (see text).

**Table 9:** LS counting results for precipitated samples of solution D as a function of settling time

LS Sample	Sample settling conditions	LS Countingresults				Assumed efficiency^c^	^36^CI Activity concentration (Bq · g^−1^)^d^	Ratio reported to measured value^e^
		Mean rate (cps · g^−1^)	*S*_m_^a^(%)	*n*^a^	*S*_p_^b^(%)			
D1	Initial (2 d–4 d)	0.03016	4.4	9	4.2	0.88 (0.025)	0.03427	1.036
	Centrifuged	0.03332	2.4	8	4.0	0.921 (0.002)	0.03618	0.982
	Further (14 d–16 d)	0.03244	2.2	15	3.0	0.899 (0.003)	0.03608	0.984
	After 21 d–33 d	0.03105	2.8	10	3.6	0.866 (0.002)	0.03586	0.990
	After 58 d–61 d	0.02988	2.3	9	3.8	0.832 (0.002)	0.03591	0.988
D2	Initial (2 d–4 d)	0.03029	3.6	10	3.7	0.88 (0.025)	0.03442	1.032
	Centrifuged	0.03417	3.8	8	3.7	0.916 (0.002)	0.03730	0.952
	Further (14 d–16 d)	0.03383	1.9	14	2.8	0.895 (0.003)	0.03780	0.939
	After 21 d–23 d	0.03080	2.9	10	3.6	0.863 (0.002)	0.03569	0.995
	After 58 d–61 d	0.02886	2.4	8	3.8	0.828 (0.002)	0.03485	1.019

a Relative standard deviation of the mean in percent for the tabulated number of measurements, *n.*

b The total statistical (Poisson) counting precision in terms of a relative standard deviation in percent, obtained in the *n* measurements. Refer to earlier footnote.

c The ^36^Cl efficiency obtained from samples of solution A with matched composition and counted after comparable settling times. Values in parentheses are estimated standard deviations for the tabulated efficiencies.

d Obtained from the LS mean count rate and the assumed ^36^Cl efficiency.

e For a reported value of 0.03551 Bq · g^−1^. See Table 1.

**Table 10:** Statistical summary of the LS counting results for solution D

Computed statistic^a^	Sample Dl	Sample D2	Both samples
Grand mean (unweighted)	0.996	0.987	0.992
*S*_m_ (%)^b^ for grand mean	1.0	1.1	1.0
Number of individual means in grand mean	5	5	10
*s*_p_ (%)^c^ for grand mean	1.6	1.5	1.1
Grand mean (weighted by number of measurements in each individual mean)	0.995	0.984	0.989
Grand mean (weighted by 1/*s*_m_^2^ in each individual mean	0.989	0.979	0.984

a Based on the data of Table 9.

b Relative standard deviation of the mean expressed in percent.

c Total statistical (Poisson) counting precision in terms of a relative standard deviation in percent obtained over all measurements. Refer to earlier footnote.

**Table 11:** Composition of the evaporated solid sourees of solutions A, B, C, and D used for the 2π*β*^−^ gas-flow-proportional counting measurements

Solution	Source identity	Mass of active solution (g)	Mass of blank NaCl solution (g)	Total mass of NaCl (mg)	Approximate ^36^Cl activity (Bq)^a^
A	Al	0.033517	0.85851	124.5	370
	A2	0.046795	1.0955	158.9	510
B	B1	0.42990	0.42070	123.3	49
	B2	0.39177	0.40158	115.0	45
C	C1	0.84273		122.2	1.7
	C2	0.87004		126.2	1.7
D	Dl	0.79823		115.7	0.03
	D2	0.88431		128.2	0.03
Blank	b		0.85921	124.6	

a Based on the reported activity concentrations given in Table 1.

**Table 12:** Summary of all source pair measurement combinations used in the determinations of their respective solution pair activity concentration ratio (or dilution factors) by 2π*β*^−^ gas-flow proportional counting

Solution pair	Total number of determinations	Source pair combinations for measurements made in counter *i*^a^							
		*i* = 2	*i* = 3	*i* = 4	*i* = 6	*i* = 7	*i* = 8	*i* = 5	*i* = 10
C/D	6	Cl/Dl	C1/D1	Cl/Dl	C2/D2	C2/D2	C2/D2		
B/D	12	Bl/Dl	Bl/Dl	Bl/Dl	B1/D2	B1/D2	B1/D2		
		B2/D1	B2/D1	B2/D1	B2/D2	B2/D2	B2/D2		
A/D	12	Al/Dl	Al/Dl	Al/Dl	A1/D2	A1/D2	A1/D2		
		A2/D1	A2/D1	A2/D1	A2/D2	A2/D2	A2/D2		
B/C	12	Bl/Cl	Bl/Cl	Bl/Cl	B1/C2	B1/C2	B1/C2		
		B2/C1	B2/C1	B2/C1	B2/C2	B2/C2	B2/C2		
A/C	12	Al/Cl	Al/Cl	Al/Cl	A1/C2	A1/C2	A1/C2		
		A2/C1	A2/C1	A2/C1	A2/C2	A2/C2	A2/C2		
A/B	28	Al/Bl	Al/Bl	Al/Bl	Al/Bl	Al/Bl	Al/Bl	Al/Bl	Al/Bl
		A1/B2	A1/B2	A1/B2	A1/B2	A1/B2	A1/B2	A1/B2	A2/B1
		A2/B1	A2/B1	A2/B1	A2/B1	A2/B1	A2/B1		
		A2/B2	A2/B2	A2/B2	A2/B2	A2/B2	A2/B2		

a Refer to Fig. 6.

**Table 13:** Summary of the numbers of 2π*β*^−^ gas-flow-proportional counting measurements performed on the evaporated solid sources

Source identity^a^	Number of meas. cycles source was mcasurcd^b^	Total number of measurements made in counter *i* for each source^b^									
		*i* = 2	*i* = 3	*i* = 4	*i* = 6	*i* = 7	*i* = 8	*i* = 1	*i* =5	*i* = 9	*i* = 10
bkgnd	18	42	45	40	42	46	43	97		64	
Al	18	5	5	7	6	5	6		30		33
A2	12	5	5	5	5	5	5				34
Bl	18	6	5	6	7	5	5		33		30
B2	12	5	5	5	5	5	5		34		
C1	6	10	11	12							
C2	6				10	11	12				
Dl	6	12	10	11							
D2	6				12	10	11				
blank	18	12	11	11	10	10	10			33	

a See Table 11.

b Refer to Fig. 6.

**Table 14:** Gas-flow proportional counting measurement results for the C/D solution pair activity concentration ratio (or dilution factor)

Counter *i*^a^	Source identity^b^	Mean count rate (cpm)	*s*_m_^c^(%)	*n*^d^	*s*_p_^c^(%)	Activity concentration ratio for C/D^f^
2	C1	38.967	0.29	10	0.17	
	bkgnd	0.2055	2.9	11	2.2	51.47
	Dl	0.9150	1.3	12	1.1	
3	C1	38.639	0.24	11	0.16	
	bkgnd	0.2310	4.3	12	2.1	53.04
	Dl	0.9139	2.1	10	1.1	
4	C1	39.072	0.22	12	0.16	
	bkgnd	0.4237	1.0	10	1.6	51.73
	Dl	1.1390	1.8	11	0.94	
6	C2	39.969	0.19	10	0.17	
	bkgnd	0.4625	3.7	11	1.5	48.70
	D2	1.2763	4.0	12	0.89	
7	C2	40.492	0.35	11	0.16	
	bkgnd	0.2443	3.4	12	2.1	52.64
	D2	1.0114	2.2	10	1.1	
8	C2	40.224	0.17	12	0.16	
	bkgnd	0.4364	2.1	10	1.4	56.62
	D2	1.1506	1.1	11	0.78	

				mean ratio		52.37
				*n*		6
				*s*_m_ (%)		2.0

a Refer to Fig. 6.

b See Table 11.

c Relative standard deviation of the mean count rate for *n* measurements expressed in percent.

d Total number of measurements in mean count rate.

e Total statistical (Poisson) counting precision in terms of a relative standard deviation in percent obtained over all *n* measurements.

f Refer to discussion in text.

**Table 15:** Gas-flow-proportional counting measurement results of all solution pairs and comparisons to the reported gravimetric dilution factors

Solution pair	Mean ratio *R*_m_^a^	*n*^b^	*s*_m_^c^(%)	Reported ratio *R*^d^	*R/R*_m_
C/D	52.37	6	2.0	56.24	1.074
B/D	2844.	12	2.8	3227.	1.135
A/D	261300.	12	4.1	308400.	1.180
B/C	54.80	12	0.65	57.39	1.047
A/C	5034.	12	1.1	5483.	1.089
A/B	91.87	28	0.17	95.55	1.040

a Mean activity concentration ratio (or dilution factor) for the solution pair.

b Total number of determinations of the ratio as given in Table 12.

c Relative standard deviation of the mean ratio for *n* determinations expressed in percent.

d See Table 1.

**Table 16:** Uncertainty analyses for the 2π*β*^−^ gas-flow-proportional counting measurement of the solution pairs

Uncertainty components and propagated uncertainties	Relative uncentainties in percent for solution pairs					
	A/B	A/C	A/D	B/C	B/D	C/D
Measurement precision, *s*_m_ of Table 15	0.17	1.1	4.1	0.65	2.8	2.0
Gravimetric aliquot determinations	0.15	0.15	0.15	0.1	0.1	0.1
Background subtractions	a	a	a	a	a	a
Radioactive decay corrections	a	a	a	a	a	a
Dead-time losses	0.2	0.2	0.2	0.02	0.02	(a)
Source *β^−^* self absorption	2.304.6^a^	2.3–4.6^a^	2.3–4.6^a^	1–2.^a^	1–2.^a^	1–2.^a^
Source positioning variations	a	0.2^a^	0.2^a^	0.2^a^	0.2^a^	0.4^a^
Combined standard uncertainty, *u*	2.3–4.6	2.6–4.7	4.7–6.2	1.2–2.1	3.0–3.5	2.3–2.9
*3u*^b^	7–14	8–14	14–19	4–6	9–10	7–9

a Assumed to be wholly, or in part, embodied in *s*_m_; or negligible.

b Corresponds to three times the combined uncertainty *u*, which is assumed to provide an uncertainty interval having a high level of confidence of roughly 95% to 99%.

## Footnotes

1 Certain commercial equipment, instruments, or materials are identified in this paper to spceify adequately the experimental procedure. Such identification does not imply recommendation or endorsement by the National Institute of Standards and Technology, nor docs it imply that the materials or equipment identified arc necessarily the best available for the purpose.

2 The total “counting error” devolves from the assumption that the radioactive decay process is Poisson distributed and that its variance (*N*) is equal to the mean number of total observed counts (*N*). Here, the combined *s*_p_ is expressed as a relative standard deviation in percent and is merely given by $100\sqrt{N_{S}+N_{B}}/(N_{S}-N_{B})$ where *N*_s_ is the total number of counts summed over all replicate measurements on a sample and *N*_B_ is the respective total number of background counts.
